# Latent profiles of problematic internet use and their associations with physical activity levels among college students: a cross-sectional study

**DOI:** 10.3389/fpubh.2026.1818418

**Published:** 2026-07-27

**Authors:** Wenzhi Peng, Zeng Zhou, Ziyi Chen

**Affiliations:** 1 Department of Physical Education Teaching and Research, Central South University, Changsha, Hunan, China; 2 College of Sports Science, Jishou University, Jishou, Hunan, China; 3 College of Physical Education and Health, Guangxi Normal University, Guilin, Guangxi, China

**Keywords:** behavioral addiction, college students, latent profiles, physical activity levels, problematic internet use

## Abstract

**Objective:**

To identify latent profiles of problematic internet use (PIU) among college students and examine their associations with physical activity (PA) levels.

**Methods:**

A stratified cluster sampling survey was conducted among 2,660 college students from central China. Problematic short video, social media, and internet game use were assessed using validated scales, and PA level was measured using the Physical Activity Rating Scale–3. Latent profile analysis was performed to identify PIU profiles. Multinomial logistic regression was used to examine the associations between PA levels and profile membership after adjusting for sociodemographic characteristics.

**Results:**

Four heterogeneous latent profiles of problematic internet use were identified among college students: the Low-risk problematic use profile (*n* = 1,630, 61.28%), the Social media–dominant problematic use profile (*n* = 304, 11.43%), the Gaming-dominant problematic use profile (*n* = 478, 17.97%), and the High-risk problematic use profile (*n* = 248, 9.32%). Multinomial logistic regression analyses showed that, compared with the Low-risk profile, male students had higher odds of membership in the Gaming-dominant profile (*OR* = 2.122, 95%*CI*:1.697–2.654) and the High-risk profile (*OR* = 1.714, 95%*CI*:1.292–2.273), but lower odds of membership in the Social media–dominant profile (*OR* = 0.767, 95%*CI*:0.598–0.983). Sophomore and junior students had significantly higher odds of membership in all three non–low-risk profiles, whereas senior students had significantly lower odds. Off-campus residence was associated with lower odds of membership in the Gaming-dominant profile (*OR* = 0.508, 95%*CI*:0.298–0.864). High-level physical activity was associated with lower odds of membership in the Social media–dominant (*OR* = 0.585, 95%*CI*:0.395–0.866), Gaming-dominant (*OR* = 0.371, 95%*CI*:0.255–0.539), and High-risk profiles (*OR* = 0.552, 95%*CI*:0.353–0.862). Moderate-level physical activity was associated with lower odds of membership only in the Gaming-dominant profile (*OR* = 0.795, 95%*CI*:0.632–0.999).

**Conclusion:**

PIU among college students exhibits substantial heterogeneity. The observed associations between PA levels and profile membership may inform targeted PIU prevention strategies.

## 1 Introduction

The college period represents a transitional stage from late adolescence to early adulthood, during which individuals gradually achieve physiological maturation, psychological independence, and the formation of autonomous lifestyles and behavioral habits. College students constitute a major population of internet users and, simultaneously, a high-risk group for internet-related problematic behaviors (1). Problematic internet use refers to a pattern of excessive and persistent internet engagement characterized by strong cravings and loss of control, resulting in significant psychological, physiological, and social functional impairments (2, 3). Problematic short video use, problematic social media use, and problematic internet game use are commonly used indicators for assessing levels of problematic internet use among college students (4). Specifically, problematic short video use is primarily manifested as compulsive consumption of short video content and emotional dependence; problematic social media use emphasizes excessive involvement in online social interactions and difficulties in behavioral control; problematic internet game use is characterized by immersion in online or offline games, accompanied by pronounced withdrawal symptoms and impairments in real-life functioning (5–7).

A growing body of research indicates that these forms of problematic internet use may exacerbate disruptions in biological rhythms among college students. For example, problematic short video use may lead to delayed sleep onset and reduced sleep quality, thereby impairing the regularity of daytime activity rhythms. Problematic social media use not only disrupts sleep rhythms but may also interfere with dietary rhythms and daily activity schedules through excessive immersion and instant feedback mechanisms. Problematic internet game use can affect multiple dimensions of biological rhythms, including irregular daily routines, delayed meals, and reduced physical activity (8, 9). Given that the college period is not only a critical stage for the rapid development of problematic internet use but also a key window for mental health intervention (10), identifying effective strategies for the prevention and regulation of problematic internet use among college students has become a central research focus across interdisciplinary fields such as education and psychology.

According to Self-Regulation Theory, the occurrence and maintenance of problematic internet use are closely associated with insufficient self-control capacity and a preference for immediate rewards. As a modifiable lifestyle behavior, physical activity (PA) is hypothesized to be associated with problematic internet use, and this relationship may potentially relate to psychological and behavioral processes linked to self-regulation, which remains to be empirically examined in future research (11, 12). Previous studies have examined the associations between physical activity and different forms of problematic internet use, with findings consistently indicating that higher levels of physical activity are significantly negatively correlated with problematic short video use, problematic social media use, and problematic internet game use (13, 14). However, there is currently no consensus regarding the strength thresholds and scope of effects of physical activity level on problematic internet use. Some studies suggest that moderate or higher levels of physical activity are sufficient to exert positive effects, whereas others indicate that only high levels of physical activity can significantly reduce the risk of addiction. Still other studies have reported that even low levels of physical activity may alleviate certain forms of problematic internet use (15–17). Therefore, a more systematic examination of the effects of different physical activity levels on the risk of problematic internet use among college students is warranted to clarify these inconsistencies.

Overall, most existing studies have adopted a variable-centered research paradigm, primarily focusing on the linear relationships between physical activity level and single forms of problematic internet use, thereby to some extent overlooking the potential heterogeneity of problematic internet use at the individual level among college students. Latent Profile Analysis (LPA) is a person-centered statistical approach that identifies latent subgroups with relatively homogeneous characteristics based on individuals’ response patterns across multiple continuous variables, enabling the identification of distinct combinations of problematic internet use among college students (18, 19). From this perspective, it is necessary to incorporate physical activity, as a modifiable lifestyle behavior, into a person-centered analytical framework.

Accordingly, the present study employed Latent Profile Analysis to identify latent profiles of problematic internet use among college students and further compared the distribution patterns of different physical activity participation levels across these latent profiles.

## 2 Methods

### 2.1 Participants

This study was conducted in November 2025 and involved four general universities in Hunan Province, China. A stratified cluster sampling method was employed. First, universities were used as the primary sampling units, and students within each university were stratified by academic grade. Subsequently, within each grade, two classes from the humanities and two classes from the sciences were randomly selected using a simple random sampling method (lottery drawing). All enrolled students in the selected classes were invited to participate in the survey.

Electronic questionnaires were distributed to students from a total of 64 classes, with 2,784 questionnaires administered. After excluding invalid questionnaires with abnormally short response times or substantial missing data, 2,660 valid questionnaires were retained, yielding an effective response rate of 95.55%. The final sample comprised 1,511 male students and 1,149 female students, with a mean age of 20.73 ± 1.86 years.

### 2.2 Instruments

#### 2.2.1 General demographic information

General demographic information included gender, grade, household registration, and on-campus residence status. Household registration was categorized as urban or rural. Urban registration referred to registration in the urban districts of prefecture-level or county-level cities, whereas rural registration referred to registration in townships or lower-level administrative regions. On-campus residence status was classified as on-campus or off-campus. Off-campus residence was defined as primarily residing at an off-campus location for at least three consecutive months prior to the survey.

#### 2.2.2 Problematic short video use

Problematic short video use was assessed using the Problematic Short Video Media Use Scale (PSVMUS) developed by Mao et al. (20). The scale consists of 13 items covering three dimensions: cognitive–behavioral changes, physiological impairment, and social viscosity. All items are rated on a 5-point Likert scale (1 = “strongly disagree” to 5 = “strongly agree”), with higher total scores indicating greater levels of problematic short video use. In the present study, the scale demonstrated good internal consistency, with a Cronbach’s *α* coefficient of 0.91.

#### 2.2.3 Problematic social media use

Problematic social media use was measured using the Social Network Addiction Tendency Scale (SNATS) developed by Wang Suping and colleagues (21). The scale includes eight items rated on a 5-point Likert scale (1 = “strongly disagree” to 5 = “strongly agree”), with higher scores indicating stronger tendencies toward problematic social media use. In this study, the scale exhibited good internal consistency, with a Cronbach’s *α* coefficient of 0.87.

#### 2.2.4 Problematic internet game use

Problematic internet game use was assessed using the Internet Game Addiction subscale of the College Student Internet Addiction Scale developed by Zhou et al. (22), namely the Internet Game Addiction Scale (IGAS). The subscale consists of eight items rated on a 5-point Likert scale (1 = “strongly disagree” to 5 = “strongly agree”), with higher scores indicating more severe problematic internet game use. In the present study, the Cronbach’s *α* coefficient for this subscale was 0.86, indicating good internal consistency.

#### 2.2.5 Physical activity level

Physical activity level was evaluated using the Physical Activity Rating Scale–3 (PARS-3), originally developed by Hashimoto Kimio and revised by Liang Deqing and colleagues (23, 24). The scale comprises three items assessing exercise intensity, frequency, and duration. Exercise intensity and frequency are each scored on a 5-point scale, ranging from 1 (“light”) to 5 (“vigorous”) for intensity and from 1 (“almost never”) to 5 (“6–7 times per week”) for frequency. Exercise duration is assessed using five ordered categories coded from 0 to 4, ranging from 0 (“≤15 min per session”) to 4 (“≥60 min per session”).

The total PARS-3 score was calculated as exercise intensity × duration × frequency, yielding a possible score range of 0–100, with higher scores indicating greater exercise volume. According to the established scoring criteria of the Chinese version of the PARS-3, scores of ≤19, 20–42, and ≥43 were classified as low, moderate, and high exercise volume, respectively. Although these criteria were developed in the 1990s, the same three-category classification criteria continue to be used in recent studies of Chinese university students (25–27). The original cutoffs were therefore retained to ensure consistency with the established PARS-3 scoring system and comparability across studies using the same instrument. In the present study, the scale demonstrated good internal consistency, with a Cronbach’s *α* coefficient of 0.89.

### 2.3 Data collection

All investigators were graduate students with relevant disciplinary backgrounds and prior survey experience. Before formal data collection, all investigators received standardized training to ensure a comprehensive understanding of the questionnaire content, response procedures, and relevant academic terminology. The survey was administered on a class-by-class basis. Prior to questionnaire distribution, investigators provided a unified explanation of the study objectives and response requirements. Students completed the questionnaires voluntarily, independently, and anonymously after being fully informed.

No intervention was made during the response process to minimize social desirability bias. After data submission, centralized quality control and data cleaning procedures were conducted, and questionnaires with invalid or patterned responses were excluded to ensure data accuracy and reliability.

### 2.4 Data analysis

All statistical analyses were performed using SPSS 29.0 and Mplus 8.3. Descriptive statistical analyses were first conducted to summarize demographic characteristics, physical activity levels, and indicators of problematic internet use among college students. Continuous variables are presented as means ± standard deviations (*M* ± *SD*), and categorical variables are presented as frequencies and percentages.

Latent Profile Analysis (LPA) was conducted in Mplus 8.3 to identify subgroups based on three indicators of problematic internet use: problematic short video use, problematic social media use, and problematic internet game use. Robust maximum likelihood estimation (MLR) was adopted for model estimation. We fitted models with one to five latent profiles. To avoid local maxima, 200 random sets of starting values and 50 final stage optimizations were specified. Following conventional LPA specifications in Mplus, indicator variances were constrained to be equal across profiles, and covariances among indicators were fixed to zero within each profile under the assumption of local independence. No missing data were identified for the LPA indicators, and all valid cases were included in the analysis. The three indicators were represented by their respective mean item scores on the original 1–5 Likert response scale. Because all indicators were measured on the same scale, no additional standardization was performed.

Model estimation started with a one-class solution, and the number of latent classes was increased sequentially. Model fit was comprehensively assessed using the Akaike Information Criterion (AIC), Bayesian Information Criterion (BIC), sample-size adjusted BIC (aBIC), entropy, the Lo–Mendell–Rubin adjusted likelihood ratio test (LMRT), and the bootstrap likelihood ratio test (BLRT). Lower AIC, BIC and aBIC values indicated better model fit. A significant result of LMRT or BLRT (*p* < 0.05) suggested that the *K*-class model outperformed the (*K* − 1)-class model. Entropy was used to evaluate the accuracy of latent profile classification. In addition, the relative size and substantive interpretability of each latent profile were considered when evaluating model adequacy. According to relevant methodological guidelines, in models with three or more profiles, any profile comprising less than approximately 10% of the total sample should be further examined in terms of its distinctiveness, stability, and substantive meaning (28).

After determining the optimal latent-profile solution, Pearson *χ*^2^ tests were conducted to compare the distributions of demographic characteristics and physical activity levels across the latent-profile groups. When an omnibus *χ*^2^ test was statistically significant, pairwise comparisons between profiles were subsequently performed with Bonferroni adjustment for multiple testing.

Prior to the multinomial logistic regression analyses, multicollinearity among the independent variables was assessed, and all variance inflation factor (VIF) values were below 5. Latent-profile membership was treated as the dependent variable, with physical activity level entered as the primary explanatory variable and gender, grade level, residence status, and household registration included as sociodemographic covariates. The Low-risk problematic use profile was specified as the reference outcome category, while female gender, freshman grade, on-campus residence, urban household registration, and low-level physical activity were specified as the respective reference categories. To further compare the associations across problematic internet use profiles, the model was rerun using the High-risk problematic use profile as the reference outcome category while retaining the same independent variables. Results are reported as odds ratios (ORs) with 95% confidence intervals (CIs). All statistical tests were two-tailed, with the significance level set at *α* = 0.05.

### 2.5 Ethical statement

This study was approved by the Biomedical Ethics Committee of Jishou University (approval number: JSDX-2023-0011), with the approval period covering the entire research process. All study procedures strictly adhered to the approved protocol, and the collected data were used solely for academic research purposes. Prior to data collection, the research team communicated with relevant administrative departments of the participating universities, providing detailed explanations of the study objectives, procedures, and ethical safeguards, and obtained written permission from the authorities. In accordance with ethical guidelines, all participants provided written informed consent after being fully informed of the study purpose, procedures, and measures for privacy and confidentiality protection.

## 3 Results

### 3.1 Common method bias

Common method bias was examined using Harman’s single-factor test. The first unrotated factor accounted for 18.67% of the total variance, which was below the critical threshold of 40% (29), indicating that common method bias was not a serious concern in the present study.

### 3.2 Participant demographics

Table 1 presents the descriptive statistics of the study sample, including Age, Grade Level, Residence Status, Household registration, scores of problematic internet use, and Physical activity level across different genders. The continuous PARS-3 score had a mean of 25.70 and a standard deviation of 22.74. Its distribution was positively skewed, with skewness = 1.440 and kurtosis = 1.621, which was consistent with the multiplicative scoring characteristics of the scale.

**Table 1:** Descriptive statistics of the sample characteristics and main variables.

Variable	Total (*n* = 2,660)	Male (*n* = 1,511)	Female (*n* = 1,149)
Age [Mean ± SD]	20.73 ± 1.86	20.84 ± 1.95	20.59 ± 1.72
Grade level [*n* (%)]			
First-year	689	376(54.6)	313(45.4)
Second-year	1,083	618(57.1)	465(42.9)
Third-year	642	357(55.6)	285(44.4)
Fourth-year	246	160(65.0)	86(35.0)
Residence status [*n* (%)]			
On-campus	2,500	1,418(56.7)	1,082(43.3)
Off-campus	160	93(58.1)	67(41.9)
Household registration [*n* (%)]			
Urban	1,630	932(57.2)	698(42.8)
Rural	1,030	579(56.2)	451(43.8)
Problematic internet use [*M* ± *SD*]			
Problematic short video use	30.61 ± 9.32	31.03 ± 9.12	30.06 ± 9.57
Problematic social media use	16.96 ± 6.22	17.09 ± 6.15	16.80 ± 6.30
Problematic internet game use	21.16 ± 5.44	21.83 ± 5.72	20.29 ± 4.91
PARS-3 score[*M* ± *SD*]	25.70 ± 22.74	25.45 ± 22.35	26.03 ± 23.25
Physical activity level [*n* (%)]			
Low-level PA	1,362	766(56.2)	596(43.8)
Moderate-level PA	904	536(59.3)	368(40.7)
High-level PA	394	209(53.0)	185(47.0)

### 3.3 Latent profiles of problematic internet use among college students

Latent Profile Analysis was used to identify latent subgroups of problematic internet use among college students, and models with one to five profiles were sequentially estimated and compared (Table 2). Although the AIC, BIC, and aBIC continued to decrease as the number of profiles increased, and the LMRT and BLRT remained significant for the five-profile model, further examination of its profile patterns indicated that the additional profile mainly represented a further severity-based subdivision of an existing profile and did not constitute a new behavioral pattern with clear theoretical distinctiveness. In addition, entropy decreased slightly from 0.922 for the four-profile model to 0.918 for the five-profile model. Therefore, considering model parsimony, profile distinctiveness, and theoretical interpretability, the four-profile solution was ultimately retained.

**Table 2:** Model fit indices for Latent Profile Analysis of problematic internet use among college students.

Model	AIC	BIC	aBIC	Entropy	LMRT(*p*)	BLRT(*p*)	Probabilities
1	17494.262	17529.579	17510.515	—	—	—	—
2	12028.939	12087.800	12056.027	0.965	<0.001	<0.001	0.63/0.37
3	10682.556	10764.961	10720.478	0.949	<0.001	<0.001	0.19/0.20/0.61
4	10477.383	10583.333	10526.141	0.922	<0.001	<0.001	0.61/0.11/0.18/0.09
5	10257.120	10386.614	10316.713	0.918	<0.001	<0.001	0.05/0.59/0.12/0.15/0.09

AIC, Akaike information criterion; BIC, Bayesian information criterion; aBIC = sample-size adjusted Bayesian Information Criterion; Entropy = classification accuracy; LMRT = *p*-value of the likelihood ratio test; BLRT = *p*-value of the bootstrap likelihood ratio test. “—” indicates that the latent class model was not applicable; therefore, entropy, LMR (p), and BLRT (p) were not available.

The classification probability matrix for the four-class solution is presented in Table 3. The average posterior probabilities of individuals being correctly classified into their respective latent profiles ranged from 85.8 to 99.5%, indicating that the four-class model demonstrated good classification accuracy and reliability.

**Table 3:** Classification quality for latent profile analysis.

Class	C1	C2	C3	C4
C1	**0.995**	0.000	0.005	0.000
C2	0.001	**0.884**	0.012	0.102
C3	0.009	0.021	**0.937**	0.033
C4	0.000	0.106	0.035	**0.858**

Bold values indicate the average posterior probabilities of correct classification for each latent profile.

**Figure 1:**
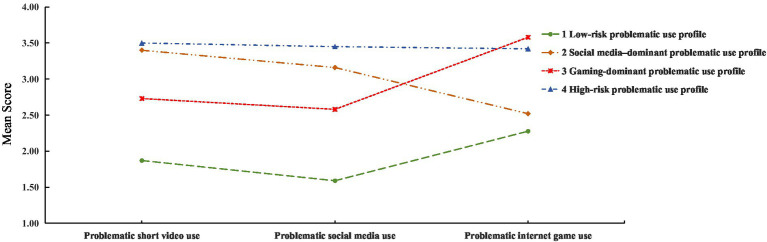
Profiles of problematic internet use across the four latent classes. Values represent class-specific unstandardized mean item scores on the original 1–5 Likert-type response scale.

Based on the relative levels of problematic short video use, problematic social media use, and problematic internet game use across the latent profiles, the classes were labeled accordingly. As shown in Figure 1, Class 1 (*n* = 1,630, 61.28%) exhibited the lowest scores across all three indicators and was therefore labeled the Low-risk problematic use profile. Class 2 (*n* = 304, 11.43%) was characterized by relatively high levels of problematic short video use and problematic social media use, accompanied by low levels of problematic internet game use, and was labeled the Social media–dominant problematic use profile. Class 3 (*n* = 478, 17.97%) showed the highest level of problematic internet game use, with relatively low levels of problematic short video use and problematic social media use, and was labeled the Gaming-dominant problematic use profile. Class 4 (*n* = 248, 9.32%) demonstrated the highest levels of problematic short video use and problematic social media use, along with relatively high levels of problematic internet game use, and was therefore labeled the High-risk problematic use profile.

### 3.4 Univariate analysis of latent profiles of problematic internet use across different physical activity levels

The results of the univariate analyses are presented in Table 4. For variables with a statistically significant omnibus *χ*^2^ test, pairwise Pearson *χ*^2^ tests were conducted between latent-profile pairs, with Bonferroni adjustment for six comparisons. Significant differences were observed among the four latent profiles of problematic internet use with respect to gender, grade level, and physical activity level (*χ*^2^ = 43.918–101.081, *p* < 0.001). In contrast, no significant differences were found across latent profiles in terms of residence status (*χ*^2^ = 6.751, *p* = 0.080) or household registration (*χ*^2^ = 6.284, *p* = 0.099).

**Table 4:** Demographic characteristics and physical activity levels across latent profiles of problematic internet use.

Variable	Low-risk	Social media–dominant	Gaming-dominant	High-risk	*χ* ^2^	*p*	Cramer’s *V*
Gender, [*n* (%)]							
Male	872(53.5)	140(46.1)	336(70.3)	163(65.7)	65.077	<0.001^bcde^	0.156
Female	758(46.5)	164(53.9)	142(29.7)	85(34.3)			
Grade, [*n* (%)]							
Freshman	477(29.3)	68(22.4)	89(18.6)	55(22.2)	101.081	<0.001^abc^	0.113
Sophomore	581(35.6)	149(49.0)	241(50.4)	112(45.2)			
Junior	368(22.6)	76(25.0)	127(26.6)	71(28.6)			
Senior	204(12.5)	11(3.6)	21(4.4)	10(4.0)			
Residence status, [*n* (%)]							
On-campus residence	1,520(93.3)	285(93.8)	461(96.4)	234(94.4)	6.751	0.080	0.050
Off-campus (commuter)	110(6.7)	19(6.3)	17(3.6)	14(5.6)			
Household registration, [*n* (%)]							
Urban	997(61.2)	190(62.5)	307(64.2)	136(54.8)	6.284	0.099	0.049
Rural	633(38.8)	114(37.5)	171(35.8)	112(45.2)			
Physical activity level, [*n* (%)]							
Low-level PA	785(48.2)	165(54.3)	282(59.0)	130(52.4)	43.918	<0.001^b^	0.091
Moderate-level PA	549(33.7)	104(34.2)	159(33.3)	92(37.1)			
High-level PA	296(18.2)	35(11.5)	37(7.7)	26(10.5)			

*χ*^2^ tests were conducted for categorical variables. Superscript letters indicate significant adjusted pairwise comparisons: a, P1 vs P2; b, P1 vs P3; c, P1 vs P4; d, P2 vs P3; e, P2 vs P4; and f, P3 vs P4. Pairwise comparisons were not interpreted when the omnibus *χ*^2^ test was nonsignificant. P1 = low-risk; P2 = social media–dominant; P3 = gaming-dominant; P4 = high-risk; PA = physical activity.

### 3.5 Multinomial logistic regression analysis of the associations between physical activity levels and latent profiles of problematic internet use

Latent profile membership was treated as the dependent variable, with the Low-risk problematic use profile as the reference category. Gender, grade level, residence status, household registration, and physical activity level were included as independent variables. Female gender, freshman grade, on-campus residence, urban household registration, and low-level physical activity were specified as the respective reference categories. The results are presented in Table 5.

**Table 5:** Multinomial logistic regression analyses of demographic characteristics and physical activity levels across latent profiles of problematic internet use.

Variable	*β*	*SE*	Wald *χ*2	*p*	*OR*(95%*CI*)
Social media-dominant problematic use profile					
Male	−0.266	0.127	4.400	0.036	0.767(0.598–0.983)
Grade					
Sophomore	0.589	0.159	13.695	<0.001	1.803(1.320–2.464)
Junior	0.390	0.182	4.617	0.032	1.477(1.035–2.109)
Senior	−0.907	0.337	7.256	0.007	0.404(0.209–0.781)
Residence status	−0.091	0.260	0.123	0.726	0.913(0.548–1.520)
Household registration	−0.050	0.130	0.148	0.700	0.951(0.737–1.228)
Physical activity level					
Moderate-level PA	−0.085	0.138	0.380	0.537	0.918(0.700–1.204)
High-level PA	−0.536	0.200	7.179	0.007	0.585(0.395–0.866)
Gaming-dominant problematic use profile					
Male	0.753	0.114	43.486	<0.001	2.122(1.697–2.654)
Grade					
Sophomore	0.799	0.141	32.236	<0.001	2.223(1.687–2.930)
Junior	0.666	0.158	17.883	<0.001	1.947(1.430–2.651)
Senior	−0.618	0.260	5.666	0.017	0.539(0.324–0.897)
Residence status	−0.677	0.271	6.235	0.013	0.508(0.298–0.864)
Household registration	−0.136	0.111	1.486	0.223	0.873(0.702–1.086)
Physical activity level					
Moderate-level PA	−0.230	0.117	3.886	0.049	0.795(0.632–0.999)
High-level PA	−0.991	0.191	26.965	<0.001	0.371(0.255–0.539)
High-risk problematic use profile					
Male	0.539	0.144	13.986	<0.001	1.714(1.292–2.273)
Grade					
Sophomore	0.500	0.177	8.005	0.005	1.649(1.166–2.332)
Junior	0.529	0.194	7.441	0.006	1.698(1.161–2.484)
Senior	−0.888	0.355	6.254	0.012	0.411(0.205–0.825)
Residence status	−0.216	0.296	0.536	0.464	0.805(0.451–1.438)
Household registration	0.262	0.139	3.553	0.059	1.299(0.990–1.706)
Physical activity level					
Moderate-level PA	0.004	0.149	0.001	0.977	1.004(0.750–1.345)
High-level PA	−0.595	0.228	6.834	0.009	0.552(0.353–0.862)

The low-risk problematic use profile was used as the reference outcome category. Female gender, freshman grade, on-campus residence, urban household registration, and low-level physical activity were specified as the reference categories. PA, physical activity; OR, odds ratio; CI, confidence interval.

The results showed that gender, grade level, residence status, and physical activity level were significantly associated with membership in at least one latent profile. Compared with the Low-risk problematic use profile, males had lower odds of membership in the Social media-dominant problematic use profile (*OR* = 0.767, 95%CI:0.598–0.983, *p* = 0.036), but higher odds of membership in the Gaming-dominant and High-risk problematic use profiles (Gaming-dominant: *OR* = 2.122, 95%CI:1.697–2.654, *p* < 0.001; High-risk: *OR* = 1.714, 95%CI:1.292–2.273, *p* < 0.001). Regarding grade level, compared with freshmen, sophomores and juniors had significantly higher odds of membership in the Social media-dominant, Gaming-dominant, and High-risk problematic use profiles, whereas seniors had significantly lower odds of membership in all three non-low-risk profiles (all *p* < 0.05). Regarding residence status, students living off campus had lower odds of membership in the Gaming-dominant problematic use profile than those living on campus (*OR* = 0.508, 95%CI:0.298–0.864, *p* = 0.013). Household registration was not associated with membership in any of the three non–low-risk profiles relative to the Low-risk profile (all *p* > 0.05).

Regarding physical activity level, compared with low-level physical activity, high-level physical activity was significantly associated with lower odds of membership in all three non-low-risk profiles (Social media-dominant: *OR* = 0.585, 95%CI:0.395–0.866, *p* = 0.007; Gaming-dominant: *OR* = 0.371, 95%*CI*:0.255–0.539, *p* < 0.001; High-risk: *OR* = 0.552, 95%*CI*:0.353–0.862, *p* = 0.009). Moderate-level physical activity was significantly associated with lower odds of membership only in the Gaming-dominant problematic use profile (*OR* = 0.795, 95%*CI*:0.632–0.999, *p* = 0.049), whereas its associations with the Social media-dominant and High-risk problematic use profiles were not statistically significant (*p* > 0.05).

To further compare the associations of physical activity levels across different problematic internet use profiles, the multinomial logistic regression model was rerun using the High-risk problematic use profile as the reference category while retaining the same sociodemographic covariates. The results are presented in Supplementary Table S1.

## 4 Discussion

### 4.1 Latent profiles of problematic internet use among college students

Previous studies have largely relied on scale scores to classify problematic internet use into simplistic dichotomies (presence vs. absence), or have assumed that higher scores merely indicate greater severity of addictive behaviors. Such approaches tend to overlook the heterogeneity within populations. By contrast, the latent profile analysis conducted in the present study enables a more nuanced understanding of behavioral participation patterns across distinct subgroups, thereby allowing for more specific and targeted recommendations.

The results indicated that problematic internet use among college students could be categorized into four latent profiles: the Low-risk problematic use profile, Social media–dominant problematic use profile, Gaming-dominant problematic use profile, and High-risk problematic use profile. Approximately 39% of college students were classified into latent profiles characterized by relatively higher levels on one or more indicators of problematic internet use, representing distinct risk subgroups that warrant preventive attention.

Specifically, 61.28% of students were classified into the Low-risk problematic use profile. This group demonstrated relatively low levels of problematic use across short video platforms, social media, and online gaming, reflecting comparatively healthy internet use patterns. Individuals in this profile may exhibit behavioral patterns consistent with stronger self-regulatory functioning and more regular daily routines. In contrast, 11.43% of students belonged to the Social media–dominant problematic use profile, characterized by high-frequency and fragmented use of social media and short video platforms, accompanied by relatively limited offline social engagement. Continuous exposure to information stimulation and emotional reinforcement may increase the risk of behavioral dysregulation and psychological burden in this group. The formation of this profile may be closely related to algorithm-driven content recommendation mechanisms of social media platforms, as well as the heightened social needs and identity development characteristic of the college stage (30, 31).

Furthermore, 17.97% of students were classified into the Gaming-dominant problematic use profile, which was marked by prominent problematic use of online games, while other forms of internet use remained relatively low. Individuals in this profile often rely on gaming to obtain a sense of achievement and relieve stress, and are more likely to experience risks such as disrupted daily routines, prolonged sedentary behavior, and reduced academic engagement. The underlying mechanisms may be associated with the highly immersive and goal-oriented characteristics of digital games (32–34).

Of particular concern, 9.32% of students were categorized into the High-risk problematic use profile, exhibiting elevated levels of problematic use across short video platforms, social media, and online gaming simultaneously. This profile reflects a compounded pattern of loss of control across multiple internet behaviors. Individuals in this group are often accompanied by pronounced time-management imbalance and disrupted daily rhythms, cumulative psychological stress, and substantially increased risks of academic and social functioning impairment. The formation of this profile may be closely linked to insufficient self-regulation capacity, escapist internet use driven by academic and interpersonal stress, and the synergistic reinforcement of high-intensity immediate feedback within digital media environments (35, 36).

Notably, these findings should also be interpreted within China’s digital and regulatory context. Chinese college students widely use domestic social media and short-video platforms such as WeChat, QQ, and Douyin (37), while China’s online gaming anti-addiction system strictly limits minors’ gaming time. This regulatory environment may have shaped participants’ gaming exposure before adulthood and should therefore be considered when generalizing these findings to other countries (38).

### 4.2 Associations between demographic factors and latent profiles

The results demonstrated that gender was significantly associated with the probability distribution of latent profiles of problematic internet use among college students. On the one hand, females were more likely to be classified into the Social media–dominant problematic use profile, which may be related to gender differences in socialization processes that emphasize emotional connection and interpersonal interaction. Compared with males, females tend to exhibit greater emotional sensitivity and stronger needs for emotional bonding and social support, and are therefore more inclined to maintain interpersonal relationships and obtain emotional feedback through social media platforms (39). In addition, females generally show higher receptivity to fragmented information and are more susceptible to the sustained attraction of emotionally framed and lifestyle-oriented content delivered by algorithmic recommendation systems, thereby forming high-frequency and persistent usage patterns (40, 41).

On the other hand, males were more likely to be classified into the Gaming-dominant problematic use profile and the High-risk problematic use profile. This tendency may be attributable to gender differences in behavioral orientation and stress-coping strategies. Males are more inclined to seek self-affirmation through competitive and confrontational activities, and the progression and reward structures inherent in digital games align well with their achievement motivation, providing rapid and continuous positive feedback (42). Moreover, when facing academic stress or emotional distress, males are more likely to adopt avoidant coping strategies, immersing themselves in prolonged gaming or multiple internet activities to alleviate real-life pressures, thereby increasing the risk of compounded problematic internet use behaviors (43).

Grade level was also significantly associated with the probability distribution of latent profiles. Compared with freshmen, sophomores and juniors were more likely to belong to non–low-risk profiles of problematic internet use, which may be attributed to the combined effects of increasing academic pressure and relative loosening of self-management. At this stage, students have largely adapted to campus life, the novelty of university has diminished, academic and interpersonal pressures accumulate, and relatively abundant discretionary time facilitates frequent internet use for emotional regulation. Meanwhile, peer imitation and reinforcement effects related to internet behaviors may further amplify addiction risk (44, 45). In contrast, seniors demonstrated lower risks of problematic internet use, possibly due to the strengthening of goal-oriented behavior driven by graduation-related demands. Their time and energy are more focused on internships, employment, or further education, which compresses time available for recreational and addictive internet use. Enhanced self-discipline, together with reduced campus supervision and social environment contraction, may jointly inhibit high-frequency internet use behaviors (46).

Residence status was also associated with the Gaming-dominant problematic use profile, with off-campus students showing lower odds of membership in the Gaming-dominant profile rather than the Low-risk profile compared with on-campus students. This association may reflect differences in daily routines and time allocation, as greater involvement in offline activities may leave less time for online gaming (47).

### 4.3 Associations between physical activity levels and latent profiles

The regression results showed that, compared with the Low-risk problematic use profile, high-level physical activity was significantly associated with lower odds of membership in the Social media–dominant, Gaming-dominant, and High-risk problematic use profiles. From a self-regulation perspective, insufficient self-control resources and a preference for immediate rewards have been proposed as potential contributors to the occurrence and maintenance of problematic internet use. Higher levels of physical activity may be associated with better executive functioning and lower levels of stress and negative emotions (48, 49). However, given the cross-sectional design of the present study, these associations should not be interpreted as evidence that physical activity prevents or reduces problematic internet use; their direction requires further examination in future longitudinal studies.

Specifically, the sense of achievement and emotional release associated with physical activity may be linked to lower reliance on reinforcement from gaming and other internet activities (50, 51). For individuals in the Social media–dominant problematic use profile, high-level physical activity may be associated with less reliance on online emotional feedback, potentially reflecting better mood and greater engagement in real-life interactions (52, 53). In addition, high-level physical activity may co-occur with more structured daily routines and time schedules, which may be associated with fewer opportunities for frequent internet use and less reinforcement of problematic behaviors (54, 55). Taken together, these findings suggest that high-level physical activity is associated with lower odds of membership in multiple non–low-risk profiles; however, the psychological and behavioral mechanisms underlying these associations remain speculative and require further investigation.

The regression results further showed that moderate-level physical activity was significantly associated with lower odds of membership in the Gaming-dominant problematic use profile, but was not significantly associated with membership in the High-risk or Social media–dominant problematic use profiles. Previous studies have associated moderate-level physical activity with better emotional states, lower psychological stress, and greater engagement in real-world activities, which may in turn be related to less reliance on prolonged and immersive online entertainment (56, 57). However, social media–dominant internet behaviors may be more strongly associated with emotional connection and immediate social feedback, with usage motives oriented toward emotional support and social identity needs. Moderate-level physical activity may therefore be less strongly associated with social media-related motivations (58, 59). Moreover, social media use is often highly fragmented and context-dependent and can be easily embedded in learning, rest, and daily life. Moderate-level physical activity may thus be less strongly associated with frequent and brief social media use patterns (60). Consequently, these findings suggest a profile-specific rather than uniform pattern in the associations between physical activity levels and problematic internet use profiles.

## 5 Strengths and limitations

### 5.1 Strengths

This study has several notable strengths. First, based on a large sample drawn from four universities in Hunan Province, China, the present study employed Latent Profile Analysis (LPA) to systematically reveal the individual-level heterogeneity of problematic internet use among college students. This person-centered analytical approach provides a more refined perspective for understanding the co-occurrence patterns of multiple problematic internet behaviors.

Second, this study simultaneously incorporated three common forms of problematic internet use-short video addiction, social networking addiction, and online gaming addiction and examined them within a unified analytical framework. This integrated approach allows for a comprehensive depiction of the overall structure of problematic internet use among college students, thereby avoiding the potential bias inherent in prior studies that focused on a single behavioral domain.

Third, this study systematically examined the differential associations between physical activity levels and distinct latent profiles of problematic internet use. The findings provide preliminary evidence that may inform differentiated health-promotion and prevention strategies for problematic internet use among college students.

### 5.2 Limitations

Several limitations of this study should be acknowledged when interpreting the findings.

First, the findings were interpreted in light of Self-Regulation Theory; however, the study did not directly measure potential mediating processes such as self-control, executive function, or stress regulation. Therefore, the proposed pathways linking physical activity and problematic internet use could not be empirically examined. Future longitudinal and experimental studies should directly assess these processes to clarify their potential roles in the observed associations.

Second, several potentially important confounding variables, such as sleep quality and academic performance, were not included in the present analyses. Existing evidence suggests that these factors are closely associated with both physical activity and problematic internet use. Their omission may have limited the interpretability of the observed associations. Future longitudinal studies are encouraged to incorporate these variables to further clarify the relationships among physical activity, problematic internet use, and related behavioral or academic outcomes.

Third, the cross-sectional design limits causal inference regarding the relationship between physical activity and problematic internet use. Reverse causality cannot be ruled out, as students with lower levels of problematic internet use may have more time and opportunities to engage in physical activity. Future longitudinal studies are needed to clarify the temporal relationship between these variables.

In addition, the sample was drawn from universities located in central China. The generalizability of the findings to other cultural contexts or educational environments remains to be verified through studies involving cross-regional and cross-cultural samples.

## 6 Conclusion

Using Latent Profile Analysis, this study identified four heterogeneous latent profiles of problematic internet use among college students: the Low-risk problematic use profile, Social media–dominant problematic use profile, Gaming-dominant problematic use profile, and High-risk problematic use profile. Gender, grade level, and residence status were differentially associated with latent-profile membership, indicating that selected sociodemographic characteristics were associated with specific problematic internet use profiles.

Furthermore, moderate-level physical activity was significantly associated with lower odds of membership in the Gaming-dominant problematic use profile, while high-level physical activity was significantly associated with lower odds of membership in the Social media–dominant, Gaming-dominant, and High-risk problematic use profiles.

Overall, the findings underscore the heterogeneity of problematic internet use among college students and suggest that the observed associations between physical activity levels and latent-profile membership may help inform stratified prevention strategies in higher education settings, although causal direction cannot be determined from the cross-sectional design.

## Data Availability

The raw data supporting the conclusions of this article will be made available by the authors, without undue reservation.
