# Supplementary material for: Latent profiles of problematic internet use and their associations with physical activity levels among college students: a cross-sectional study
Source: Front Public Health. 2026 Jul 27;14:1818418. doi: 10.3389/fpubh.2026.1818418 (PMC13453751; doi:10.3389/fpubh.2026.1818418)
Supplement: Supplementary file 1 [file Data_Sheet_1.PDF]

Supplementary Table S1. Multinomial logistic regression analysis using the High-risk profile as the reference category

Note: The reference categories were female for gender, freshman for grade, on-campus residence for residence status, urban for household registration, and low-level PA for physical activity level. PA, physical activity; *OR*, odds ratio; *CI*, confidence interval.

Variable	*β*	*SE*	Wald χ^2	*P*	*OR* (95%*CI*)
**Low-risk problematic use profile**					
Male	-0.539	0.144	13.986	<0.001	0.583 (0.440-0.774)
Grade					
Sophomore	-0.500	0.177	8.005	0.005	0.606 (0.429-0.858)
Junior	-0.529	0.194	7.441	0.006	0.589 (0.403-0.862)
Senior	0.888	0.355	6.254	0.012	2.431 (1.212-4.878)
Residence status	0.216	0.296	0.536	0.464	1.242 (0.695-2.217)
Household registration	-0.262	0.139	3.553	0.059	0.770 (0.586-1.010)
Physical activity level					
Moderate-level PA	-0.004	0.149	0.001	0.977	0.996 (0.744-1.333)
High-level PA	0.595	0.228	6.834	0.009	1.813 (1.161-2.832)
**Social media-dominant problematic use profile**					
Male	-0.805	0.177	20.677	<0.001	0.447 (0.316-0.633)
Grade					
Sophomore	0.089	0.221	0.163	0.687	1.093 (0.709-1.687)
Junior	-0.139	0.247	0.317	0.573	0.870 (0.536-1.412)
Senior	-0.018	0.475	0.001	0.970	0.982 (0.387-2.490)
Residence status	0.125	0.366	0.117	0.732	1.134 (0.553-2.323)
Household registration	-0.312	0.175	3.179	0.075	0.732 (0.519-1.031)
Physical activity level					
Moderate-level PA	-0.090	0.186	0.231	0.631	0.914 (0.634-1.318)
High-level PA	0.059	0.286	0.042	0.837	1.061 (0.606-1.858)
**Gaming-dominant problematic use profile**					
Male	0.214	0.168	1.623	0.203	1.238 (0.891-1.720)
Grade					
Sophomore	0.299	0.207	2.090	0.148	1.348 (0.899-2.022)
Junior	0.137	0.228	0.361	0.548	1.147 (0.734-1.791)
Senior	0.270	0.422	0.411	0.521	1.311 (0.573-2.996)
Residence status	-0.461	0.371	1.542	0.214	0.631 (0.305-1.306)
Household registration	-0.398	0.160	6.199	0.013	0.672 (0.491-0.919)
Physical activity level					
Moderate-level PA	-0.234	0.169	1.914	0.166	0.791 (0.568-1.102)
High-level PA	-0.396	0.278	2.034	0.154	0.673 (0.390-1.160)

Using the High-risk profile as the reference category, high-level PA was associated with higher odds of Low-risk profile membership (*OR*=1.813, 95%*CI*:1.161-2.832, *P*=0.009), whereas moderate-level PA was not (*OR*=0.996, 95%*CI*:0.744-1.333, *P*=0.977). Neither PA level significantly differentiated the Social media-dominant or Gaming-dominant profiles from the High-risk profile. Rural household registration was associated with lower odds of Gaming-dominant rather than High-risk profile membership (*OR*=0.672, 95%*CI*:0.491-0.919, *P*=0.013); the other residence-status and household-registration comparisons were nonsignificant.
